# Chemically defined conditions for long-term maintenance of pancreatic progenitors derived from human induced pluripotent stem cells

**DOI:** 10.1038/s41598-018-36606-7

**Published:** 2019-01-24

**Authors:** Shuhei Konagaya, Hiroo Iwata

**Affiliations:** 1 Institute for Frontier Medical and Life Sciences, Kyoto University, 53 Kawahara-cho, Shogoin, Sakyo-ku, Kyoto, 606-8507 Japan; 2 Research Promotion Institution for COI Site, Kyoto University, Yoshida-honmachi, Sakyo-ku, Kyoto, 606-8501 Japan; 3 The “Compass to Healthy Life” Research Complex Program, RIKEN, 6-7-1 Minatojima-Minamimachi, Chuo-ku, Kobe, 650-0047 Japan

## Abstract

Large numbers of hormone-releasing cells, approximately 10^9^ endocrine cells, are required to treat type I diabetes patients by cell transplantation. The SOX9-positive pancreatic epithelium proliferates extensively during the early stages of pancreatic development. SOX9-positive pancreatic epithelium is thought to be an expandable cell source of β cells for transplantation therapy. In this study, we attempted to expand pancreatic progenitors (PPs: PDX1^+^/SOX9^+^) derived from four human iPSC lines in three-dimensional (3D) culture using a chemically defined medium and examined the potential of the derived PPs to differentiate into β-like cells. PPs from four human iPSC lines were maintained and effectively proliferated in a chemically defined medium containing epidermal growth factor and R-spondin-1, CHIR99021, fibroblast growth factor-7, and SB431542. PPs derived from one iPSC line can be expanded by more than 10^4^-fold in chemically defined medium containing two of the fives, epidermal growth factor and R-spondin-1. The expanded PPs were also stable following cryopreservation. After freezing and thawing, the PPs proliferated without a decrease in the rate. PPs obtained after 50 days of culture successfully differentiated into insulin-positive β-like cells, glucagon-positive α-like cells, and somatostatin-positive δ-like cells. The differentiation efficiency of expanded PPs was similar to that of PPs without expansion culture.

## Introduction

Pluripotent stem cells (PSCs), such as embryonic stem cells and induced pluripotent stem cells (iPSCs), have been suggested as sources for cell replacement therapy for type I diabetes^1,2^. Large numbers of hormone-releasing cells, approximately 10^9^ cells, are required to treat a type I diabetes patient by cell transplantation^3,4^. Although PSCs can undergo unlimited expansion, several weeks are required to prepare β-like cells from PSCs. Additionally, obtaining reproducible differentiation efficiency between batches remains difficult. Fully differentiated β-like cells rarely proliferate^5^, while immature cells such as pancreatic progenitors (PPs) were reported to be capable of self-renewal on feeder cells and differentiation into endocrine^6^ and exocrine lineages^7^.

Various progenitors have been identified in pancreatic development^8^. During the early stages of pancreatic development, SRY-box 9 (SOX9)-positive pancreatic epithelium proliferates extensively and undergoes branching morphogenesis^9^. More committed cells, such as neurogenin 3 (NEUROG3, NGN3)-positive endocrine progenitors, exhibit a limited proliferation capacity^10^. Although these results were obtained using mice and mouse cells, SOX9-positive PPs derived from human pluripotent stem cells may be useful as an expandable cell source of β-like cells for transplantation therapy. Additionally, the risk of teratoma formation can be reduced by culturing cells for a long period *in vitro* before transplantation, because contamination with undifferentiated PSCs and progenitors of other lineages can be monitored and removed during PP expansion culture.

Recently, pancreatic organoid culture was introduced to prepare models for pancreatic development and pancreatic cancer^11–13^. PPs isolated were from ductal tissues collected from the mouse and human pancreas, embedded in Matrigel, and cultured in the presence of epidermal growth factor (EGF) and R-spondin-1 (RSPO1)^11,12^. RSPO1 is known to induce the proliferation of intestinal, hepatic, and pancreatic progenitors by regulating Wnt signaling^13^. While it was also reported that PPs, which proliferate extensively in organoid culture, rarely differentiate into β cells after organoid culture^11^. Additionally, Matrigel, an animal-derived extracellular matrix, was used as a culture scaffold^11,12^. For the clinical use of PSC-derived β-like cells, chemically defined culture conditions should be developed to prevent contamination with xenogeneic proteins.

In this study, we attempted to expand PPs (PDX1^+^/SOX9^+^) derived from four human iPSC lines in three-dimensional (3D) culture using chemically defined medium, and examined their cryopreservation and potential to differentiate into β-like cells.

## Results

### Proliferation of PPs derived from hiPSC in chemically defined medium containing EGF and RSPO1

**Figure 1:**
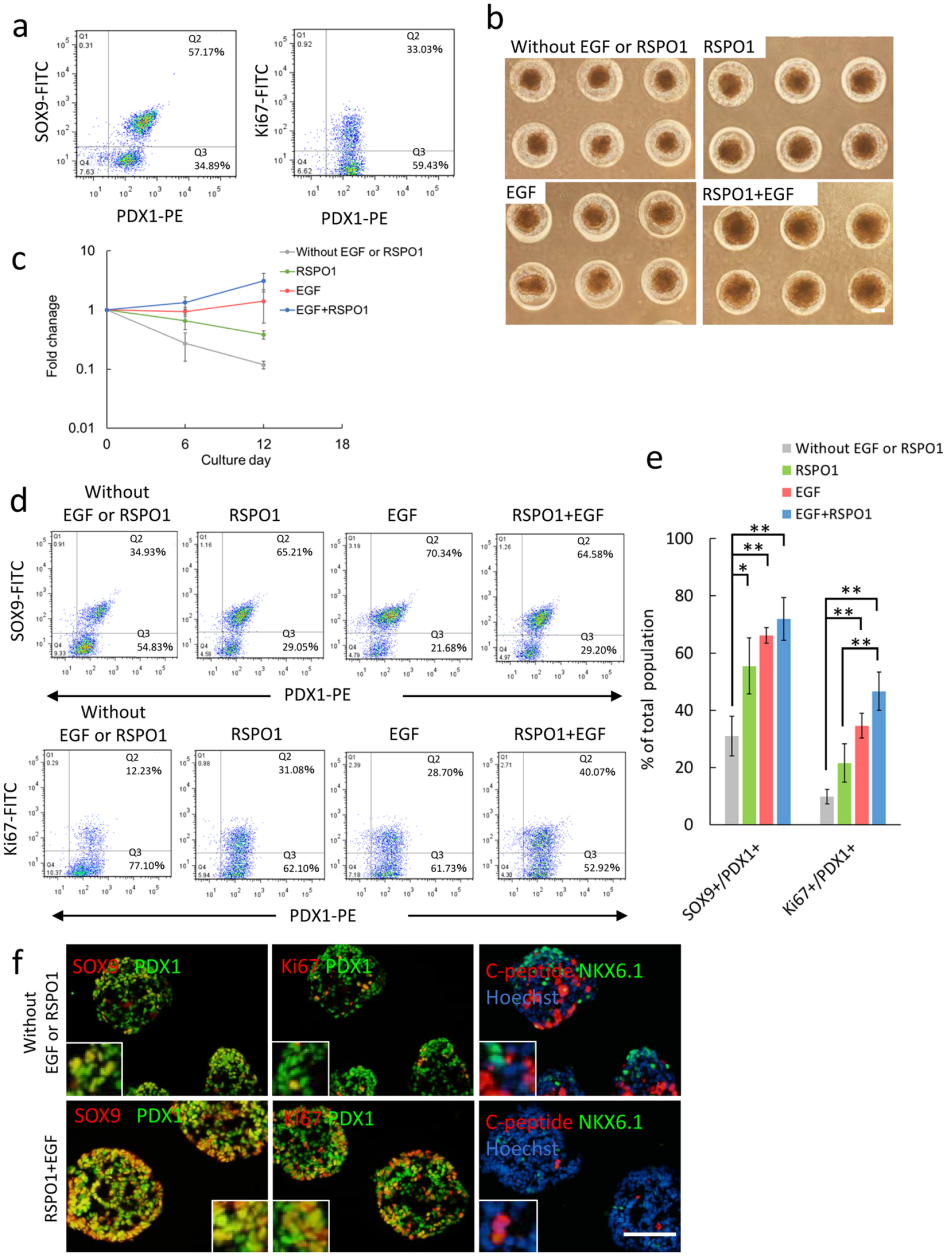
EGF and RSPO1 synergistically induced the proliferation of hiPSC-PPs in short-term culture. (**a**) Representative results of flow cytometry analyses for PDX1 and SOX9 (right panel) or PDX1 and Ki67 (left panel). hiPSCs 253G1 line were differentiated into PPs (Stage 4) and then stained with antibodies. (**b**) Phase-contrast images of cell aggregates of human iPSC (253G1 cell)-derived PPs. Cell aggregates were cultured for 6 days in the presence of EGF and/or RSPO1. (**c**) Fold-change in cell number within 12 days (mean ± standard deviation, n = 3, *p < 0.01, Tukey’s HSD test). On day 6, cell aggregates were subcultured. The results were expressed as fold-change relative to the initial cell numbers. (**d**) Representative results of flow cytometry analyses for PDX1 and SOX9 (upper panel) or PDX1 and Ki67 (bottom panel). Cell aggregates were cultured for 6 days and then stained with antibodies. (**e**) Summary of flow cytometry analyses (mean ± standard deviation, n = 3, **p < 0.01, *p < 0.05, Tukey’s HSD test). (**f**) Immunofluorescent micrographs of thin sections of cell aggregates cultured in microwells with or without EGF + RSPO1 for 6 days. Cells were immunologically stained with antibodies targeting pancreatic and cell proliferation markers. Cell nuclei were stained with Hoechst 33258. Inserts: high magnification images of single and double stained cells. Scale bars = 100 μm.

PPs were derived from the human iPSC 253G1 line using the stepwise differentiation protocol established by Rezania *et al*.^14^. Stage 4 cells, representing the pancreatic endoderm, were used as a cell source for expansion culture (Supplementary Fig. 1). On day 2 of stage 4, most cells were PDX1-positive (>90%); approximately half of these cells were SOX9^+^ cells, while approximately 30% of the cells expressed Ki67, a marker of proliferating cells (Fig. 1a). The culture medium was replaced with a newly developed chemically defined PP growth medium (PP-GM) containing EGF, RSPO1, and additional small molecules such as SANT1, LDN 193189, retinoic acid (Supplementary table 1). PPs were cultured and expanded as cell aggregates in the chemically defined medium using microwell plates composed of agarose hydrogel. Although we observed cell proliferation when the culture medium contained either EGF or RSPO1 in some extent, the combination of EGF and RSPO1 was more effective for promoting cell proliferation (Fig. 1b and c). The ratios of PDX1^+^/SOX9^+^ progenitors to PDX1^+^/Ki67^+^ proliferating pancreatic cells in complete PP-GM (EGF + RSPO1) were higher than those in medium containing either EGF or RSPO1 (Fig. 1d–f). While addition of EGF alone promoted the proliferation of PDX1^+^/SOX9^+^ progenitors, some differentiated into PDX1^+^/NKX6.1^+^ endocrine progenitors (Supplementary Fig. 2).

Without EGF and RSPO1, PPs showed slow proliferation. The cell number was decreased through dissociation and re-aggregation (Fig. 1c) and some cells differentiated into NKX 6.1- and/or C-peptide-positive endocrine cells (Fig. 1f). In addition to growth stimulation by EGF and RSPO1, 3D culture, cell aggregates, was necessary for the remaining PPs. When PPs were two dimensionally cultured on Geltrex-coated surfaces in the presence of EGF and RSPO1, the cells expanded over several days; however, the cells gradually differentiated into exocrine and ductal cells, after which the cell number was dramatically decreased (Supplementary Fig. 3).

### Some characters of PPs after large expansion of cell numbers

**Figure 2:**
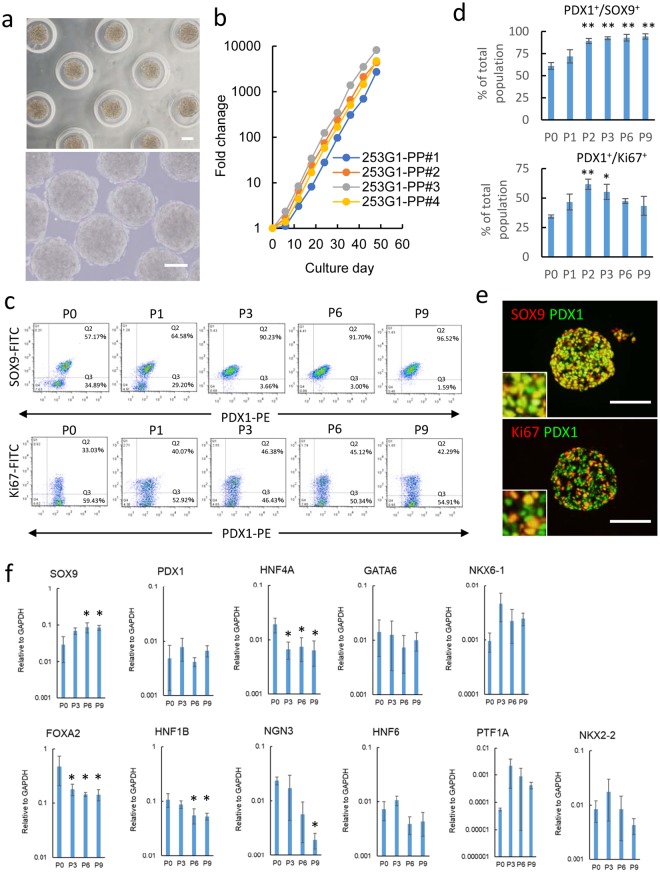
Long-term maintenance of PPs derived from hiPSCs (253G1 cells). (**a**–**e**) 253G1 cell-derived PPs were maintained in PP-GF for long-term analysis. Black circles indicate each passaging point. Cells were subcultured every 6 days. (**a**) Phase-contrast images of cell aggregates during long-term culture (passage 5). Top panel: image of cell aggregates cultured in microwell, bottom panel: image of cell aggregates retrieved from microwell. (**b**) Fold-change in cell number (n = 4). Circles indicate each passaging point. The results were expressed as fold-change relative to the initial cell numbers. (**c**) Representative results of flow cytometry analyses for PDX1 and SOX9 (upper panel) or PDX1 and Ki67 (bottom panel) though long-term culture. P0–P9: passage numbers. (**d**) Summary of flow cytometry analyses (mean ± standard deviation, n = 3, **p < 0.01, *p < 0.05, compared to P0, Tukey’s HSD test). (**e**) Fluorescent micrograph of thin sections of cell aggregates after long-term culture. Cells were stained with antibodies targeting pancreatic markers. Inserts: high-magnification images of single and double stained cells. Scale bars = 100 μm. (**f**) Summary of qPCR analyses. Expression levels were normalized to GAPDH expression (mean ± standard deviation, biological replicates, n = 3). PPs before expansion (P0) and after three (P3), six (P6), and nine passages (P9) were used for the study. *p < 0.05 compared to P0, Tukey’s HSD test.

Next, we assessed the expansion ability of iPSC-derived PPs. Every 6 days, cell aggregates of PPs were dissociated into single cells, reseeded into microwell plates, and cultured in PP-GM (Fig. 2a). As shown in Fig. 2b, the cell number increased by approximately 10,000-fold over 50 days. Percentage of PDX1^+^/SOX9^+^ progenitors in the cells gradually increased and reached approximately 95% after several passages (Fig. 2c and d). In an earlier stage of our expansion culture (P0–P1), NKX 6.1^+^/PDX1^+^ endocrine progenitor cells also formed aggregates (Supplementary Fig. 2). However, the cells showed minimal proliferation in PP-GM and the cell population gradually decreased. The ratio of proliferating pancreatic cells, PDX1^+^/Ki67^+^ cells, was approximately 50% throughout the culture period (Fig. 2c and d). After long-term expansion culture, most cells were positive for PDX1, SOX9, and FOXA2 (Fig. 2e and Supplementary Fig. 4). In contrast, the expanded cells showed minimal expression of the endocrine markers NGN3, NeuroD1, or NKX 6.1 (Supplementary Fig. 4). Quantitative polymerase chain reaction (qPCR) analyses revealed no significant changes in the expression of PP markers, such as PDX1, SOX9, PTF1A, and NKX2.2 (Fig. 2f). These results suggest that PPs can maintain their differentiated states under 3D culture in PP-GM for a prolonged time. Karyotypes of the PPs were analyzed, which were normal after a long-term culture (Supplementary Fig. 5). Additionally, expanded PPs could be cryopreserved. The post-thaw cell viability was 87.5 ± 4.3% (n = 5). After freezing and thawing, the cryopreserved PPs proliferated without a decreased rate (Supplementary Fig. 6).

### Differentiation of PPs into endocrine cells after large expansion of cell numbers

**Figure 3:**
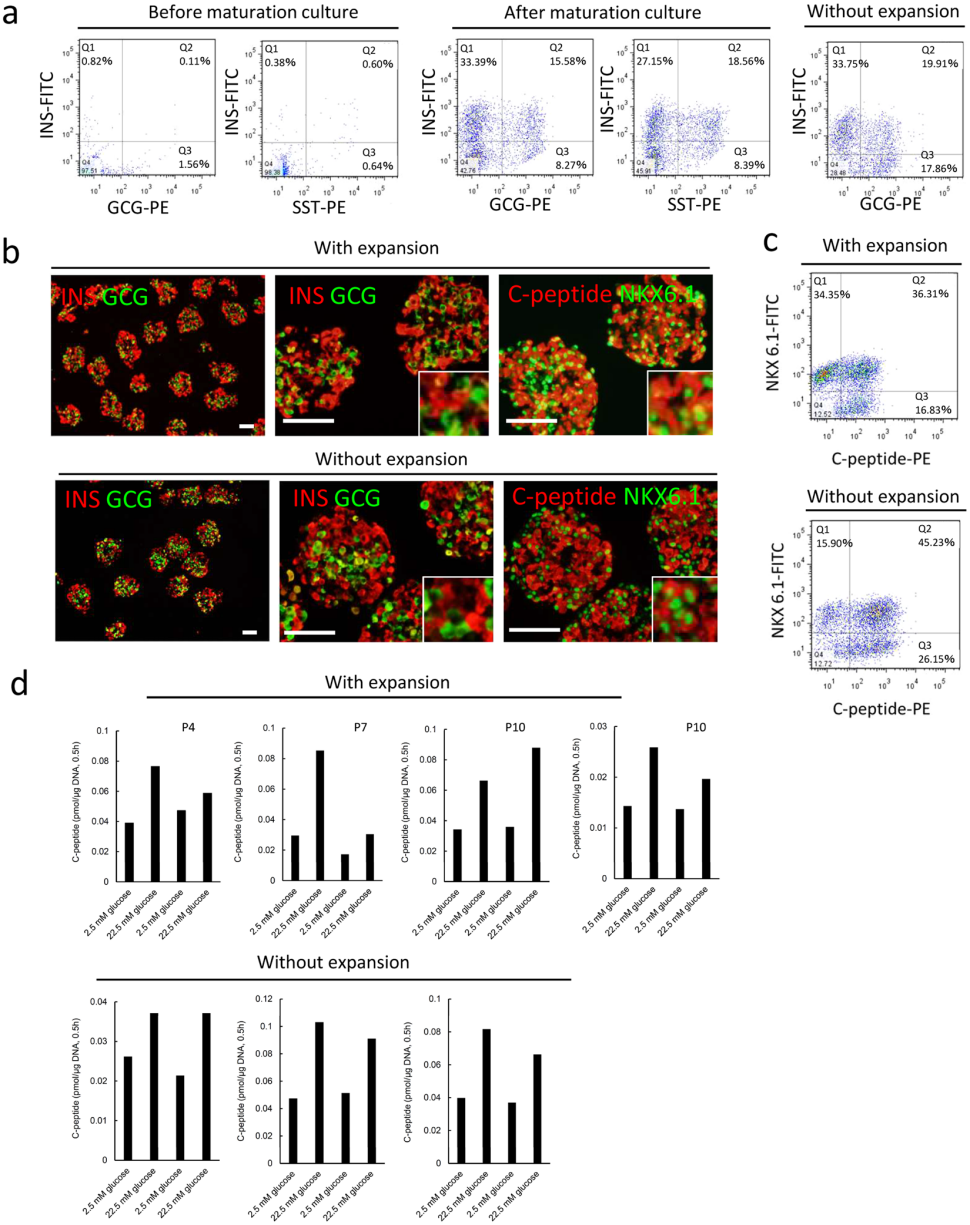
Differentiation into endocrine cells. After long-term culture, PPs were differentiated into endocrine cells. (**a**) Representative results of flow cytometry analyses for pancreatic hormones. Left: results of before maturation culture, middle: results of after maturation culture, right: results of after maturation culture without expansion culture. Cells were stained with antibodies targeting endocrine markers. INS: insulin, GCG: glucagon, SST: somatostatin. (**b**) Fluorescent micrograph of thin sections of cell aggregates after maturation culture. Cells were stained with antibodies targeting endocrine markers. Inserts: high-magnification images of single and double stained cells. Scale bars = 100 μm. (**c**) Representative results of flow cytometry analyses for C-peptide and NKX6.1. After maturation culture, the cells were fixed and stained. (**d**) C-peptide secretion from differentiated cells in response to 2.5 or 22.5 mM glucose. With (P4: n = 1, P7: n = 1, P10: n = 2) or without (P0, n = 3) expansion culture, cells were differentiated into endocrine cells and exposed to glucose solution.

The expanded PPs were examined for their ability to differentiate into endocrine cells, particularly β-like cells, following the stepwise differentiation protocol established by Rezania *et al*.^14^. After 3 weeks of maturation culture, the PPs successfully differentiated into insulin-positive β-like cells, glucagon-positive α-like cells, and somatostatin-positive δ-like cells (Fig. 3a). Although a large number of INS^+^/GCG^+^ and INS^+^/SST^+^ double-positive cells were generated from expanded PPs, similar numbers of polyhormonal cells were observed among cells derived from PPs without expansion culture (Fig. 3a–c). The differentiation efficiency of expanded PPs was similar to that of PPs without expansion culture (Fig. 3b and c). In the end stage of the maturation culture, most cells were no longer proliferating (Supplementary Fig. 7). The endocrine cells gained the ability to alter C-peptide release in response to changes in glucose concentrations (Fig. 3d). Even after cryopreservation, PPs maintained ability to differentiate into insulin-positive β-like cells (Supplementary Fig. 6d).

### Expansion of PPs derived from other hiPSC lines

**Figure 4:**
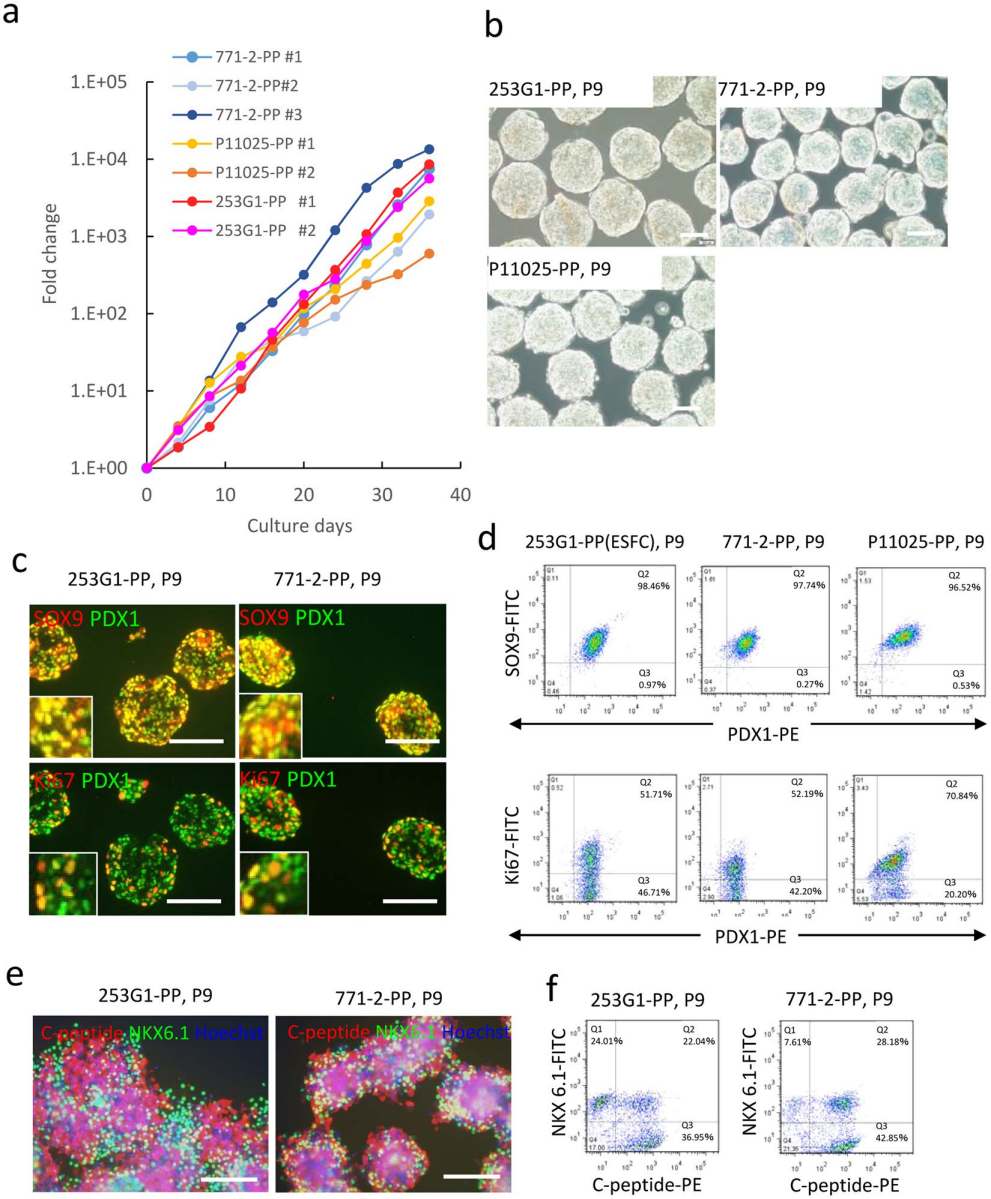
Expansion of PPs derived from various hiPSC lines. 253G1, RPChiPS771-2, and P11025 cell-derived PPs were cultured in improved PP-GM (EGF + RSPO1 + CHIR99021 + FGF7). (**a**) Fold-change in cell numbers (253G1: n = 2, 771-2: n = 3, P11025: n = 2). (**b**) Phase-contrast images of PPs after long-term culture (P9). (**c**) Fluorescent micrographs of thin sections of cell aggregates after long-term culture. Cells were stained with antibodies targeting PDX1/SOX9 or PDX1/Ki67. (**d**) Representative results of flow cytometry analyses for PDX1/SOX9 (upper panel) or PDX1/Ki67 (bottom panel) after long-term culture. Scale bars = 100 μm. (**e**,**f**) Maturation of various hiPSC-derived PPs into endocrine cells. PPs were expanded in the improved PP-GM (EGF + RSPO1 + CHIR99021 + FGF7) for long-term culture (P9) and differentiated into endocrine cells. (**e**) Fluorescent micrograph of cell aggregates after maturation culture. After maturation culture, cells were cultured on iMatrix-511-coated substrate for 2 days and stained with antibodies targeting endocrine markers. Cell nuclei were stained with Hoechst 33258. Scale bars = 100 μm. (**f**) Representative results of flow cytometry analyses for C-peptide and NKX6.1.

We examined the potential of PP-GM to expand PPs derived from some other iPSC lines. For PPs derived from 771-2 and P11025 hiPSCs, stimulation with EGF and RSPO1 was not sufficient for large expansion. PPs tended to differentiate and form acinar-like structures (Supplementary Fig. 8). New additives to the culture medium were examined to increase the ability to stimulate PP proliferation. A glycogen synthase kinase 3 inhibitor, CHIR99021, was added to increase the activation of Wnt signaling, as β-catenin is required for maximal proliferation in PPs^15^. Fibroblast growth factor 7 (FGF7), which is frequently added to media for pancreatic differentiation culture^14^, was also added to the PP-GM. Additionally, SB431542, an inhibitor of the SMAD pathway, was added. Using the newly developed culture medium, PPs derived from 771-2 and P11025 and 253G1 hiPSCs effectively proliferated as shown in Fig. 4a and b. The doubling times of the PPs in the improved PP-GM were slightly shorter, and the number of PPs increased by approximately 3-fold within 4 days of culture. Most of the expanded PPs were positive for PDX/SOX9 and approximately 60% of cells were positive for PDX1/Ki67 after 40 days culture (Fig. 4c and d). The PPs maintained their potential to differentiate into endocrine cells as shown in Fig. 4e and f after 40 days culture.

## Discussion

In the present study, we established new culture conditions for the large-scale preparation of iPSC-derived PPs. The conditions of cell aggregate culture in chemically defined medium enabled us to selectively expand the PPs. EGF and RSPO1 synergistically induced the proliferation of PPs derived from the hiPS 253G1 line (Fig. 1). However, the two cytokines were not sufficient for expanding PPs derived from the 771-2 and P11025 hiPSCs lines (Supplementary Fig. 8). We improved the culture medium by adding CHIR99021, FGF-7, and SB431542. The PPs derived from these hiPSCs were successfully expanded in the medium (Fig. 4).

Using our PP-expansion methods, the percent of PDX1^+^/SOX9^+^ progenitors in PPs increased and reached approximately 95% after several passages (Fig. 2). However, the expression of individual markers of pancreatic development was highly variable over time and between replicates (Fig. 2f). The population of expanded PPs may have contained a mixture of different pancreatic progenitors. Further studies such as transcriptome analysis by RNA-seq are needed to characterize the expanded cells.

Some researchers have reported the expansion of intermediate cells for pancreatic differentiation from PSCs^16–20^. Cells in the early stages of differentiation, such as definitive endoderm and foregut stem cells, are relatively easy to expand in chemically defined medium^16,17^. Some groups reported expansion of PPs on organ-matched mesenchyme or 3T3 fibroblasts^18–20^. These studies demonstrated quite large-scale expansion of PPs with an approximately 10^15^-fold change. However, the possibility of contamination of unknown substances derived from feeder cells and feeder cells themselves become major obstacles to the clinical application of insulin secreting cells derived from iPS / ES cells. In addition, the expanded cells were not well-characterized. In this study, we demonstrated only 10^4^-fold expansion of PPs derived from the human iPSC 253G1 line in chemically defined medium and we did not observe decrease in the proliferation rate in the late stage subculture, that is, passage 9. After this series of experiments, we started to expand PPs derived from other hiPS lines. We observed over 10^8^-fold expansion of PPs (passage 16). In the presence of EGF and RSPO1. We can obtain sufficient number of PPs by the method developed in this study. These results suggest that our method is suitable to prepare PPs which will be useful in preparation insulin releasing cells for clinical usage.

In recent years, some groups reported the organoid culture of PPs or pancreatic ductal cells derived from the rodent pancreas^11–13^. We also found that PPs effectively proliferated in the 3-D culture. While they gradually differentiated into exocrine and ductal cells in adherent culture and showed minimal expansion (Supplementary Fig. 3). Stem cell niches, comprised of cell-cell interactions and/or autocrine/paracrine signaling, may support the maintenance and proliferation of PPs. Clonal expansion of PPs is important to obtain well characterized cells for their clinical application and also required to study development of pancreas. Unfortunately, the organoid culture of PPs is not suitable for clonal expansion of PPs. We did not examine to expand PP cells clonally.

For the clinical application of iPSC-derived PPs, further studies are required to prepare well-characterized endocrine cells. A substantial number of polyhormonal cells were generated from expanded PPs (Fig. 3) in this study. However, PPs without expansion culture also generated polyhormonal cells. These results suggest that the generation of polyhormonal cells may not have resulted from PP expansion but rather because of the characteristics of the iPS cell line used in this study. The iPS/ES cell line should be carefully selected to prepare high-quality endocrine cells. Additionally, the functions of β-like cells from PPs such as the control of insulin release in response to glucose levels should be carefully examined *in vitro* and *in vivo*.

## Methods

### Human iPSC culture

Three human iPSC lines, i.e., 253G1^21^ (RIKEN Cell Bank, Ibaraki, Japan), P11025 (Takara Bio, Inc., Shiga, Japan), and RPChiPS771-2 (ReproCELL Inc., Kanagawa, Japan), were used in this study. 253G1 cells were maintained on SNL 76/7 cells (ECACC, Salisbury, UK) as a feeder layer as described previously^22^. P11025 cells were maintained using a Cellartis DEF-CS 500 Culture System (Takara Bio). RPChiPS771-2 cells were maintained on a Geltex (Life Technologies, Carlsbad, CA, USA)-coated culture surface using StemFit AK02N (Ajinomoto Co., Inc., Tokyo, Japan).

### Preparation of agarose microwell plates

A mold (Microtissues, Inc., Providence, RI, USA) was used to produce hydrogel plates with 256 wells (16 × 16 wells, 400 μm diameter) as described previously^23^. Hot 2.5% agarose solution (SeaKem GTG; Lonza, Basel, Switzerland) in saline was added to the molds and cooled to form a gel. Each agarose hydrogel plate was equilibrated in Dulbecco’s modified Eagle’s medium/F12 (Sigma-Aldrich, St. Louis, MO, USA) overnight before use. A homemade mold (made of polydimethylsiloxane, 1000 wells, 800 μm diameter, 800 μm depth) was also used to prepare agarose microwell plates, and the 1000-well plates were used for long-term culture experiments.

### Differentiation into the pancreatic linage

The 253G1 cell line was used to examine the feasibility of pancreatic progenitor (PP) culture unless otherwise mentioned. The cells were subcultured onto a Geltex-coated culture surface and cultured in Essential 8 (E8) medium (Life Technologies) for 3–4 days. At 70–80% confluence, the iPSCs were treated with TrypLE (Life Technologies) for 3 min at room temperature. Single cells suspended in the culture medium were collected, centrifuged at 1000 rpm for 5 min, and then resuspended in E8 medium containing 10 μM Y-27632 (Wako Pure Chemistry, Osaka, Japan). 253G1 cells were directed to differentiate into the pancreatic linage via a previously reported method^14^, with some modifications, as shown in Supplementary Figure 1. The cells were seeded into 256 agarose microwells at a density of 2.5 × 10^3^ cells/well (6.4 × 10^5^ cells/plate). The cells were cultured for 24 h to induce aggregation. The culture medium was changed daily according to the following time schedule.

BM1: MCDB131 medium (Life Technologies) supplemented with 1.5 g/L sodium bicarbonate, 0.5% fat-free bovine serum albumin (BSA; Wako), 2 mM GlutaMAX Supplement (Life Technologies), and 10 mM d-glucose (Nacalai Tesque, Kyoto, Japan).

BM2: MCDB131 medium (Life Technologies) supplemented with 1.5 g/L sodium bicarbonate, 2% fat-free BSA (Wako), 1/200 ITS-X supplement (Insulin, Transferrin, Selenium, Ethanolamine Solution; Life Technologies), 2 mM GlutaMAX Supplement (Life Technologies), and 20 mM d-glucose (Nacalai Tesque).

Stage 1 (3 days): BM1, 3 μM CHIR99021 (Millipore, Billerica, MA, USA), and 100 ng/mL activin A (R&D Systems, Minneapolis, MN, USA). CHIR99021 was added to the culture medium only for the first day.

Stage 2 (3 days): BM1 and 50 ng/mL FGF-7 (PeproTech, Rocky Hill, NJ, USA) + 0.25 mM ascorbic acid (Sigma).

Stage 3 (2 days): BM2, 50 ng/mL FGF-7 (PeproTech), 0.25 mM ascorbic acid (Sigma), 0.25 μM SANT-1 (Wako), 100 nM LDN 193189 (Wako), 1 μM retinoic acid (Sigma-Aldrich), and 200 nM TPB ((2 S,5 S)-(E,E)-8-(5-(4-(trifluoromethyl)phenyl)-2,4-pentadienoylamino)benzolactam, Millipore).

Stage 4 (2–4 days): BM2, 2 ng/mL FGF-7 (PeproTech), 0.25 mM ascorbic acid (Sigma), 0.25 μM SANT-1 (Wako), 200 nM LDN 193189 (Wako), 100 nM retinoic acid (Sigma-Aldrich), and 100 nM TPB (Millipore).

Other iPSC lines (P11025 and RPChiPS771-2) were differentiated into PPs by adherent culture. Dissociated hiPSCs were subcultured on a Geltex-coated culture surface at density of 1.2–1.5 × 10^5^ cells/cm^2^ and differentiated to PPs using the culture medium and culture periods mentioned above.

### Aggregate culture for expansion of pancreatic progenitors and maturation into endocrine cells

After differentiation stage 4, the culture medium was replaced with PP-GM: MCDB131 medium supplemented with 1.5 g/L sodium bicarbonate, 2% fat-free BSA, 1/200 ITS-X supplement, 2 mM GlutaMAX Supplement, 20 mM d-glucose, 0.25 μM SANT-1, 200 nM LDN 193189, 100 nM retinoic acid, 50 ng/mL EGF (Wako), and 200 ng/mL R-spondin-1 (R&D Systems). The culture medium was replaced every other day. After 6 days of floating culture, cell aggregates were collected and treated with TrypLE for 3 min at room temperature. The cell aggregates were then dissociated into single cells by pipetting. The cells were centrifuged at 1000 rpm for 5 min and then resuspended in PP-expansion medium containing 10 μM Y-27632 (ROCK inhibitor). Cells were manually counted by the trypan blue exclusion method. The cells were seeded into 256 agarose microwells at a density of 1.0 × 10^3^ cells/well (2.6 × 10^5^ cells/plate). The cell aggregates were subcultured every 6 days. Y-27632 was added to culture medium only on the first day after subculturing.

In some experiments (see Fig. 4), 50 ng/mL FGF7, 4.5 μM CHIR99021, and 10 μM SB431542 were added in the PP-GM, and PPs were subcultured every 4 days.

For maturation into endocrine cells, the culture medium was changed according to the following time schedule.

Stage 5 (3 days): BM2, 0.25 μM SANT-1 (Wako), 200 nM LDN 193189 (Wako), 50 nM retinoic acid (Sigma-Aldrich), 1 μM T3 (Sigma-Aldrich), 10 μM ALK5 inhibitor (Enzo Life Science, Farmingdale, NY, USA), 10 μM ZnSO_4_, and 10 μg/mL heparin (Nacalai Tesque).

Stage 6 (7 days): BM2, 200 nM LDN 193189, 1 μM T3, 10 μM ALK5 inhibitor, 0.1 μM GS inhibitor XX (Merck Millipore), 10 μM ZnSO_4_, and 10 μg/mL heparin.

Stage 7 (7–14 days): BM2, 1 μM T3, 10 μM ALK5 inhibitor, 10 μM Trolox (Enzo Life Science), 2 μM R428 (Selleckchem, Houston, TX, USA), 1 mM N-Cys (Sigma-Aldrich), 10 μM ZnSO_4_, and 10 μg/mL heparin.

### Immunostaining

Antibodies used for immunohistochemistry are shown in Supplementary Table 2. Cells were fixed with 4% paraformaldehyde in phosphate-buffered saline (PBS) for 30 min at room temperature and then sequentially soaked in 5% sucrose/PBS for 12 h at 4 °C, 10% sucrose/PBS for 12 h at 4 °C, and 20% sucrose/PBS for 12 h at 4 °C. The cells were embedded in Tissue-Tek (Sakura Finetechnical Co., Ltd., Tokyo, Japan) and frozen. Frozen specimens were prepared at a thickness of 6 μm.

The specimens were treated with 0.2% Triton X-100 solution for 15 min at room temperature to permeabilize the cells and then treated with Blocking One Reagent (Nacalai Tesque) for 90 min to block the nonspecific adsorption of antibodies. Antibody solutions were applied to the specimens and incubated for 2 h at room temperature. After washing with PBS containing 0.05% Tween 20, the specimens were treated with fluorescently labeled secondary antibodies (1:500 dilution) for 1 h at room temperature and then washed with PBS containing 0.05% Tween 20. The cell nuclei were counterstained with 1 μg/mL Hoechst 33258 (Dojindo Laboratories, Kumamoto, Japan). The localization of secondary antibodies was analyzed with a fluorescent microscope (BX51 TRF; Olympus Optical Co., Ltd., Tokyo, Japan).

### Flow cytometry analysis

Cell aggregates were dissociated into single cells as described above and fixed with 4% paraformaldehyde in PBS. The cells were stained with fluorescein isothiocyanate- and phycoerythrin-labeled antibodies. The populations of fluorescently active cells were analyzed using a Guava EasyCyte Mini Flow cytometer (Millipore) equipped with a 488-nm diode laser. Data from the control experiments were used to set the threshold.

### qPCR

Cells were collected in a centrifuge tube and washed with cold PBS. cDNA was prepared by reverse transcription using Cells-to-CT Kits (Thermo Fisher Scientific, Waltham, MA, USA). Real-time PCR was carried out using the StepOne Real-Time PCR System (Life Technologies). Reaction mixtures (20 μL) containing Power SYBR Green PCR Master Mix (Life Technologies), 200 ng cDNA template, 50 nM sense primer, and 50 nM antisense primer were subjected to PCR. The primers^23,24^ used for amplification are listed in Supplementary Table 3. The expression level of each gene was normalized to the expression of the gene encoding glyceraldehyde 3-phosphate dehydrogenase (GAPDH). Three batches of PPs (253G1 cells) were used for this experiment, and the results were averaged.

### Glucose stimulation test and C-peptide enzyme-linked immunosorbent assay (ELISA)

A total of 256 aggregates were collected from the agarose microwell plates and pre-incubated in 2.5 mM glucose/Krebs-Ringer’s buffer (KRB) for 1 h. The collected aggregates were exposed sequentially to 2.5 and 22.5 mM glucose in KRB for 1 h at 37 °C. Next, the cells were depolarized with 30 mM KCl in KRB for 30 min. The supernatants were collected, and the C-peptide concentration of each solution was determined using an Ultrasensitive C-peptide ELISA Kit (Mercodia, Uppsala, Sweden) according to the manufacturer’s instructions. The C-peptide values for each sample were normalized to the DNA quantity of stimulated cells.

### Statistical analysis

Data are provided as the means ± standard deviations of at least three independent experiments. All statistical calculations were performed using JMP software (SAS Institute, Inc., Cary, NC, USA).

## Electronic supplementary material

SUPPLEMENTARY INFO
