# Supplementary material for: Chemically defined conditions for long-term maintenance of pancreatic progenitors derived from human induced pluripotent stem cells
Source: Sci Rep. 2019 Jan 24;9:640. doi: 10.1038/s41598-018-36606-7 (PMC6345937; doi:10.1038/s41598-018-36606-7)
Supplement: Supplementary file 1 — SUPPLEMENTARY INFO [file 41598_2018_36606_MOESM1_ESM.pdf]

Supplementary Information

Chemically-defined conditions for long-term maintenance of pancreatic progenitors derived from human induced pluripotent stem cells

Shuhei Konagaya[1] and Hiroo Iwata[1,2,3]

[1]Institute for Frontier Medical and Life Sciences, Kyoto University, 53 Kawahara-cho, Shogoin, Sakyo-ku, Kyoto 606-8507, Japan

[2] Research Promotion Institution for COI Site, Kyoto University, Yoshida-honmachi, Sakyo-ku, Kyoto 606-8501, Japan

[3]The "Compass to Healthy Life" Research Complex Program, RIKEN, 6-7-1 Minatojima-Minamimachi, Chuo-ku, Kobe 650-0047, Japan

*Correspondence should be addressed to Prof. Hiroo Iwata

E-mail: iwata@frontier.kuoyo-u.ac.jp

Phone: +81-75-751-4119

Fax: +81-75-751-4646

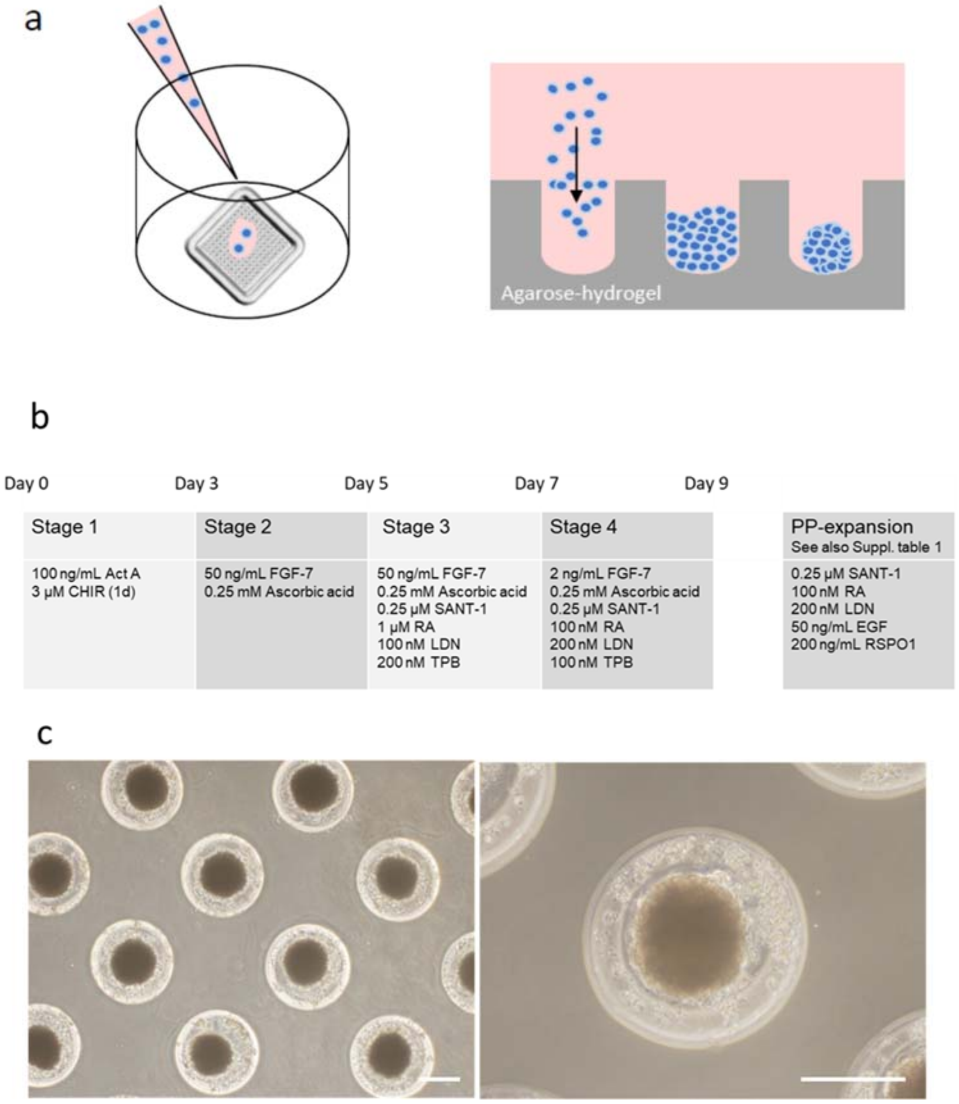

Supplementary Figure 1. Differentiation of human 253G1 iPSCs into PPs. a: Illustration of cell aggregate culture using agarose microwell plates. Cell aggregates were prepared from human iPSCs using an agarose microwell plate and differentiated into PPs. b: Time schedule of differentiation culture. ROCKi, ROCK inhibitor; Act A, activin A; CHIR, CHIR99021; FGF-7, fibroblast growth factor 7; SANT-1, sonic hedgehog signaling pathway inhibitor ; LDN, LDN 193189; RA, retinoic acid; TPB, protein kinase C activator. c: Phase-contrast images of human iPSC (253G1) aggregates formed and cultured for 9 days in an agarose microwell plate to induce differentiation into PPs. Scale bars = 200 µm.

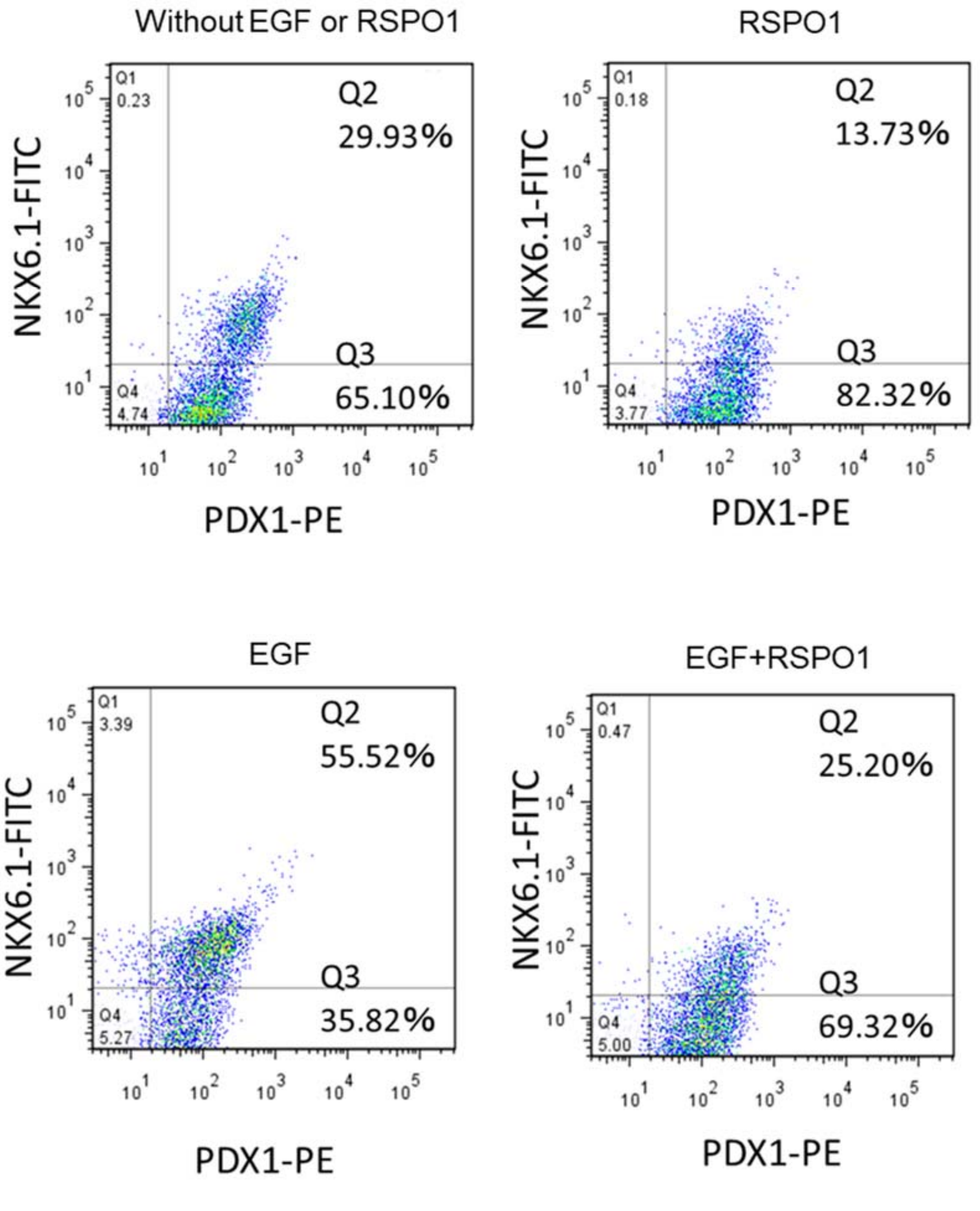

Supplementary Figure 2. Representative results of flow cytometry analyses for PDX1 and NKX6.1. Cell aggregates were cultured with or without cytokines for 6 days and then stained with antibodies.

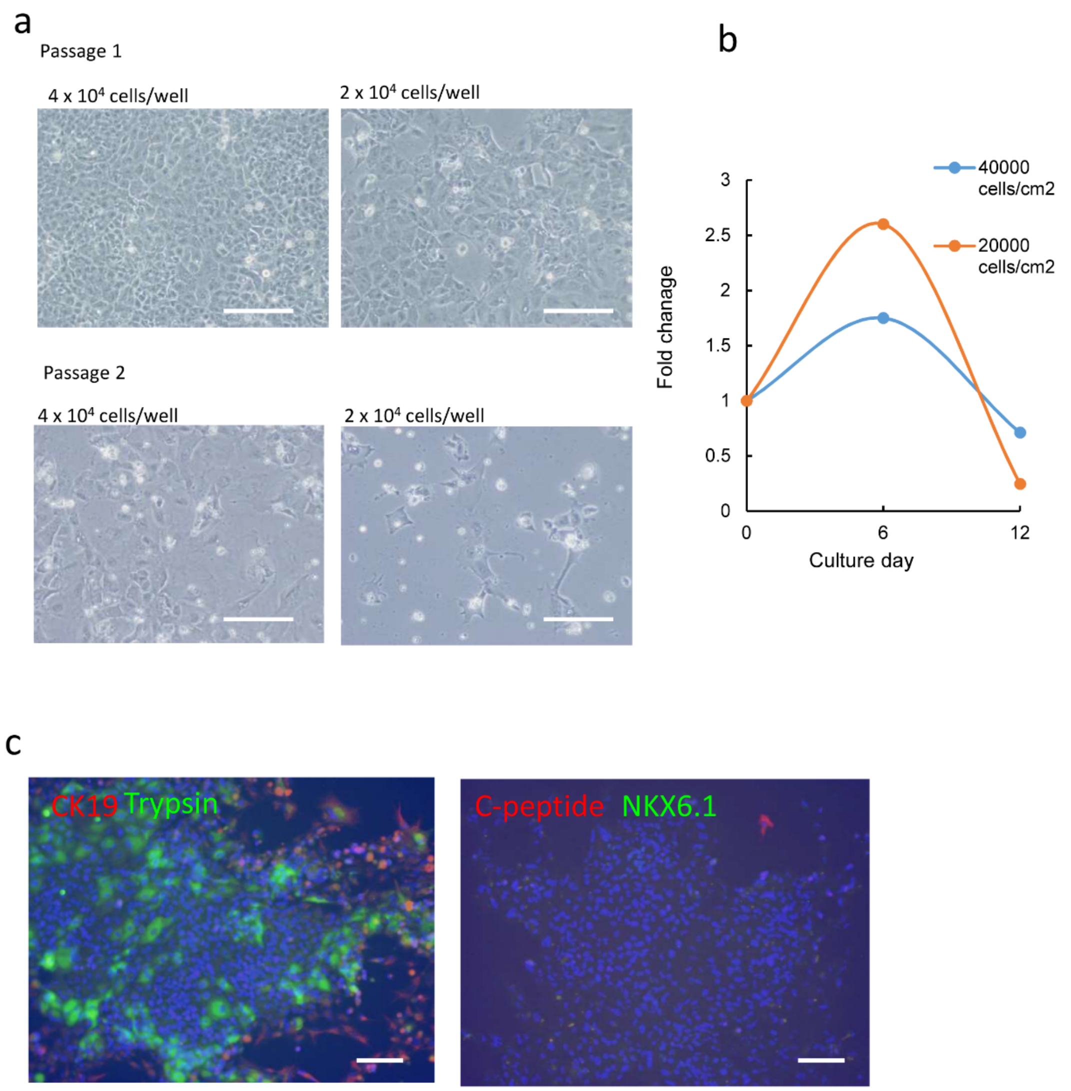

Supplementary Figure 3. Adherent culture of PPs. 253G1 cell-derived PPs were cultured on the Geltrex-coated surface at a density of 2×10^4 cells/cm^2 or 4×10^4 cells/cm^2. Cells were cultured in PP-GM and subcultured on day 6. a: Phase-contrast images of cells on day 6 (upper) and day 12 (lower). b: Fold changes in cell number. c: Fluorescent micrographs of cells cultured on the Geltrex-coated surface. Cells were stained with antibodies targeting exocrine and duct markers (left) or endocrine markers (right). Cell nuclei were stained with Hoechst 33258. Scale bars = 100 µm.

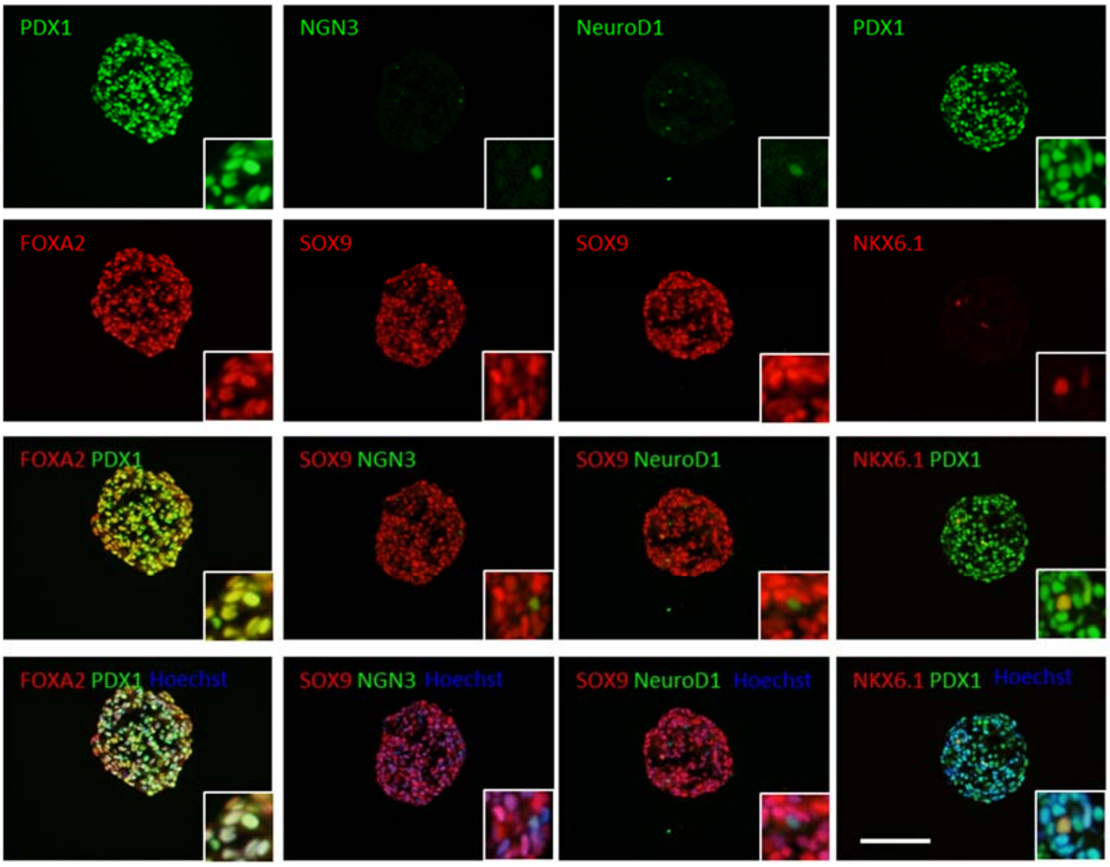

Supplementary Figure 4. Long-term maintenance of PPs. Fluorescent micrograph of thin sections of PPs (253G1 cells) after long-term culture (P9). Cells were stained with antibodies targeting endocrine markers. Cell nuclei were stained with Hoechst 33258. Scale bars = 100 μm.

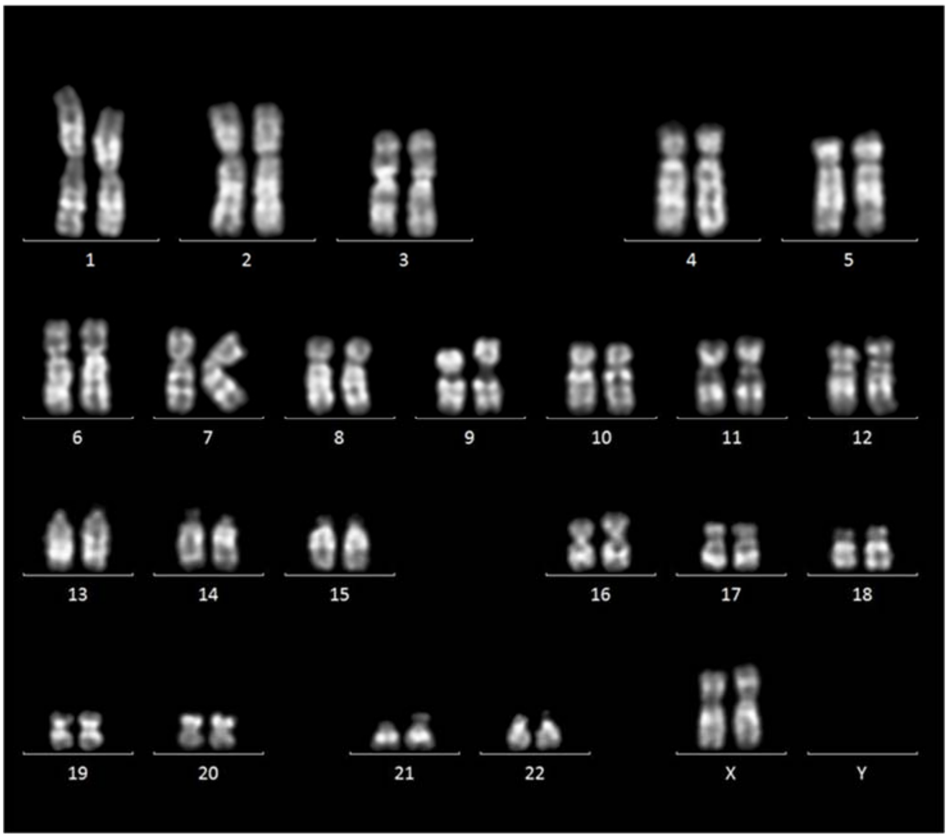

Supplementary Figure 5. Karyotype analysis of PPs. PPs derived from 253G1 iPS cells were were expanded in PP-GM for long-term (P7). The cells were fixed with Carnoy's solution (3:1 (v/v) methanol/glacial acetic acid) at room temperature.

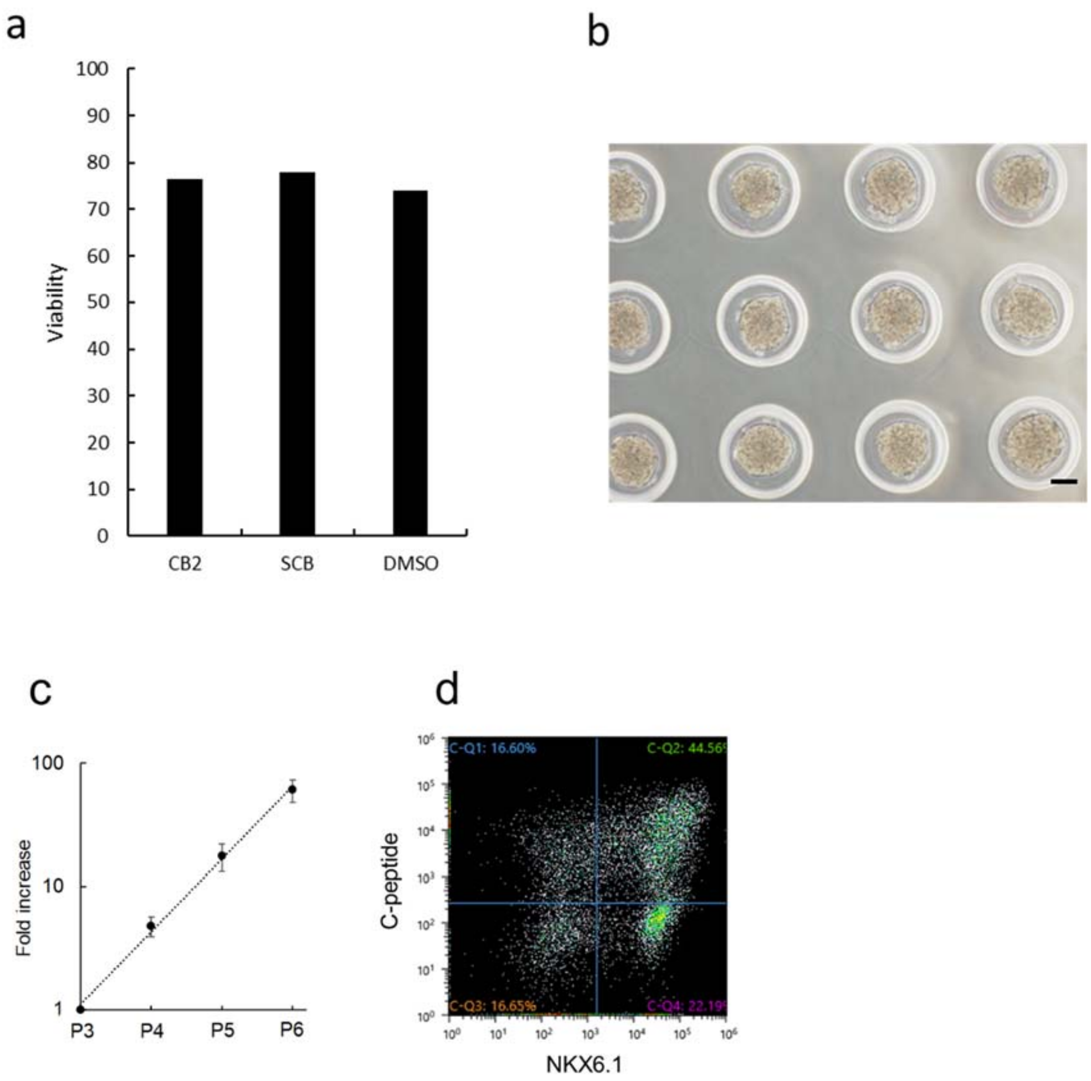

Supplementary Figure 6. Cryopreservation of PPs. PPs (P3 or P5) were cryopreserved using commercially available cryoprotective agents. a: viability of PPs soon after thawing. CB2, SCB and DMSO indicate that PPs were cryopreserved by using Cell banker 2 (CB2, Nippon Zenyaku Kogyo), stem cell banker (SCB, Nippon Zenyaku Kogyo Co., Ltd., Fukushima, Japan), PP-GM with 10 % DMSO, respectively. b: Phase-contrast images of cell aggregates prepared from freezing-thawing PPs after 6 days culture. Scale bars = 100 µm. c: Fold changes in cell number of PPs after freezing and thawing. d: Representative results of flow cytometry analyses for C-peptide and NKX6.1. After thawing, PPs were expanded in PP-GM. At passage 9, that is, passage 6 after thawing, PPs were differentiated into β-like cells.

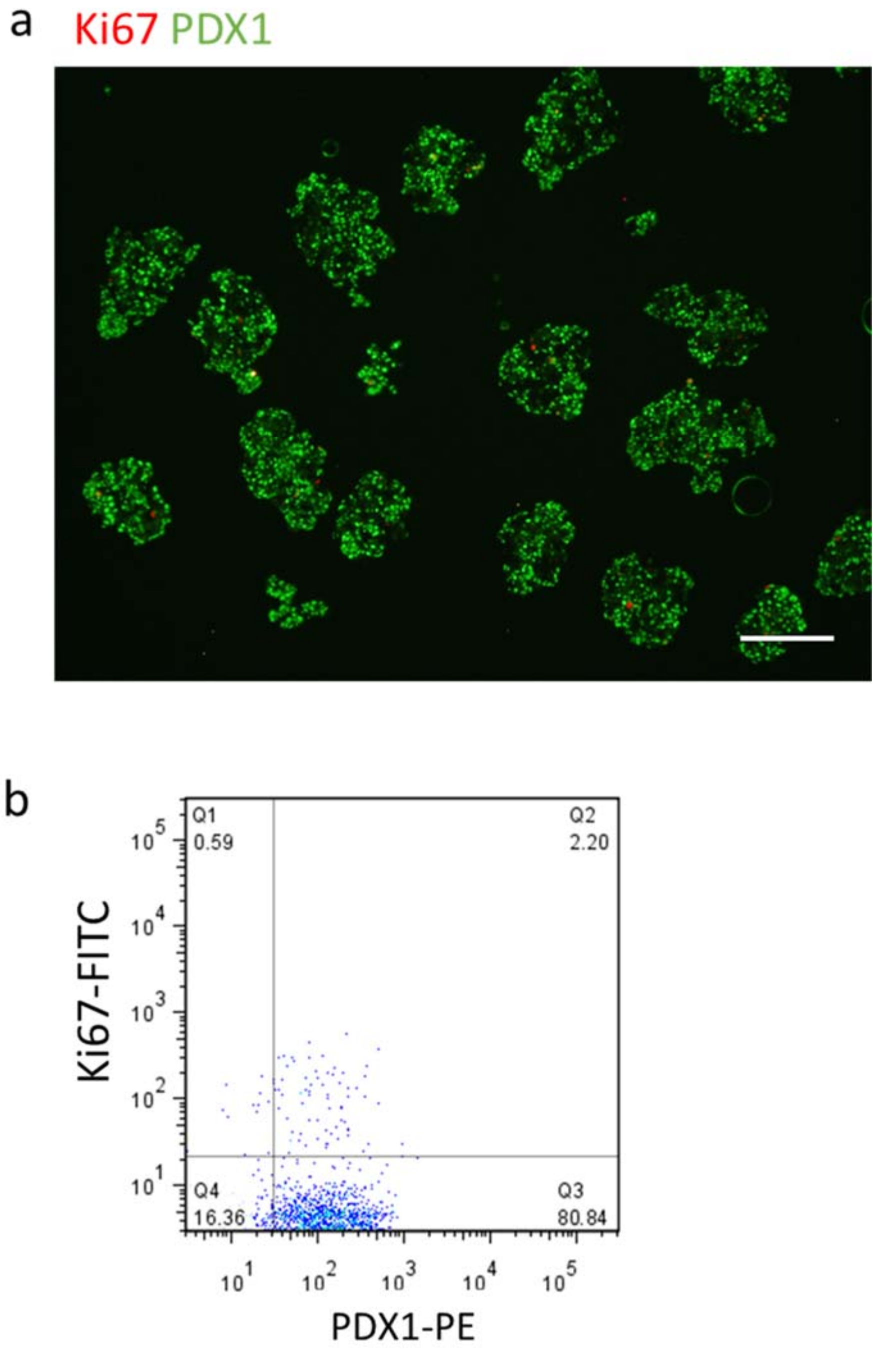

Supplementary Figure 7. Decrease in proliferating cells after differentiation culture. a: Fluorescent micrograph of thin sections of cell aggregates after maturation culture. Cells were stained with antibodies targeting PDX1 and Ki67. Scale bars = 200 µm. Cell aggregates were b: Representative results of flow cytometry analyses for PDX1 and Ki67.

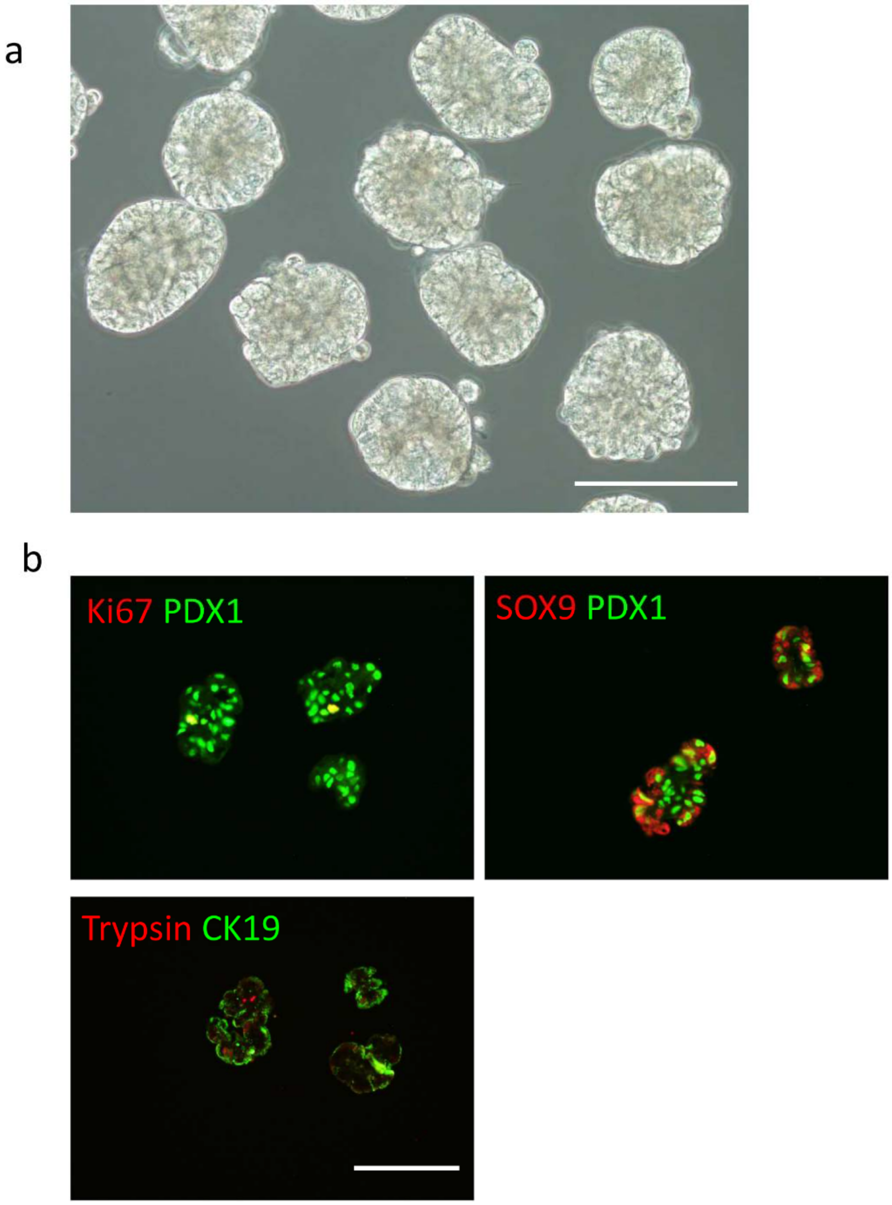

Supplementary Figure 8. Exocrine differentiation of P11025 hiPSC-derived PPs in PP-GM (without CHIR99021, FGF7, and SB431542). a: Phase contrast images of cells cultured in PP-GM for 4 days. Cells exhibited an acinar cell-like structure. b: Fluorescent micrograph of thin sections of cell aggregates. Cells were stained with antibodies against exocrine (Trypsin) and ductal cell (CK19) markers. Scale bars = 100 μm.

Supplementary table 1. Media formulation of PP-GM.

PP-GM

Components	Final concentration
Base medium	
MCDB131	
sodium bicarbonate	2.68 g/L
GlutaMAX	2 mM
D-glucose	20 mM
fat-free BSA,	2 %
ITS-X supplement	1/200
SANT-1	0.25 μM
LDN 193189	200 nM
retinoic acid	100 nM
EGF	50 ng/mL
R-spondin 1	200 ng/mL

improved PP-GM

Components	Final concentration
Base medium	
MCDB131	
sodium bicarbonate	2.68 g/L
GlutaMAX	2 mM
D-glucose	20 mM
fat-free BSA,	2 %
ITS-X supplement	1/200
SANT-1	0.25 μM
LDN 193189	200 nM
retinoic acid	100 nM
EGF	50 ng/mL
R-spondin 1	200 ng/mL
FGF7	50 ng/mL
CHIR99021	4.5 μM
SB431542	10 μM

Supplementary table 2. Antibodies used for immunofluorescence staining.

ANTIGEN	SPECIES	SOURCE	DILUTION
PDX1	Goat	R&D systems, Minneapolis, MN	1:200
SOX9	Rabbit	Merck Millipore, Billerica, MA	1:200
NKX 6.1	Rabbit	LifeSpan BioSciences, Inc., Seattle, WA	1:100
NGN3	Sheep	R&D systems, Minneapolis, MN	1:200
C-peptide	Mouse	Sanbio monosan, Uden, Netherlands	1:200
Insulin	Guinea pig	Abcam, Cambridge, UK	1:100
Insulin	Rabbit	Cell Signaling Technology, Danvers, MA	1:200
Glucagon	Mouse	Sigma-Aldrich, St. Louis, MO	1:200
Somatostatin	Goat	Santa Cruz Biotechnology, Inc., Santa Cruz, CA	1:100
Synaptophysin	Rabbit	Novus Biologicals, LLC, Littleton, CO	1:100
Trypsin	Sheep	R&D systems, Minneapolis, MN	1:200
CK19	Mouse	Dako, Denmark	1:200
Ki67	Rabbit	Cell Signaling Technology, Danvers, MA	1:200
Alexa 488 Anti-goat IgG	Donkey	Life Technologies, Carlsbad, CA	1:500
Alexa 488 Anti-rabbit IgG	Goat	Life Technologies, Carlsbad, CA	1:500
Alexa 488 Anti-Guinea pig IgG	Goat	Life Technologies, Carlsbad, CA	1:500
Alexa 594 Anti-mouse IgG	Goat	Life Technologies, Carlsbad, CA	1:500
Alexa 594 Anti-rabbit IgG	Donkey	Life Technologies, Carlsbad, CA	1:500
Alexa 594 Anti-Sheep IgG	Goat	Life Technologies, Carlsbad, CA	1:500
FITC- Anti-rabbit IgG	Donkey	BioLegend, San Diego, CA	1:500
FITC- Anti-goat IgG	Goat	BD Biosciences, San Jose, CA	1:500
PE- Anti-goat IgG	Donkey	Life Technologies, Carlsbad, CA	1:500
PE- Anti-mouse IgG	Goat	BD Biosciences, San Jose, CA	1:500

Supplementary table 3. Primer sequences used for qPCR analysis.

Primer name	Sequence
SOX17 forward	GGCGCAGCAGAATCCAGA
SOX17 reverse	CCACGACTTGCCCAGCAT
FOXA2 forward	GGGAGCGGTGAAGATGGA
FOXA2 reverse	TCATGTTGCTCACGGAGGAGTA
GATA6 forward	CTCAGTTCCTACGCTTCGCAT
GATA6 reverse	GTCGAGGTCAGTGAACAGCA
HNF4A forward	CATGGCCAAGATTGACAACCT
HNF4A reverse	TTCCCATATGTTCCTGCATCAG
HNF1B forward	TCACAGATACCAGCAGCATCAGT
HNF1B reverse	GGGCATCACCAGGCTTGTA
HNF6 forward	CGCTCCGCTTAGCAGCAT
HNF6 reverse	GTGTTGCCTCTATCCTTCCCAT
SOX9 forward	AGTACCCGCACTTGCACAAC
SOX9 reverse	ACTTGTAATCCGGGTGGTCCTT
HES1 forward	AGTGAAGCACCTCCGGAAC
HES1 reverse	TCACCTCGTTCATGCACTC
PTF1A forward	GAAGGTCATCATCTGCCATCG
PTF1A reverse	GGCCATAATCAGGGTCGCT
LGR5 forward	TTTGGACAAGGGAGACCTGGAGAA
LG5R forward	AGAGGAGAAGGACAAGAAAGCCACA
PDX1 forward	AAGTCTACCAAAGCTCACGCG
PDX1 reverse	GTAGGCGCCGCCTGC
NKX 2.2 forward	GGCCTTCAGTACTCCCTGCA
NKX 2.2 reverse	GGGACTTGGAGCTTGAGTCCT
NKX 6.1 forward	CACACGAGACCCACTTTTTC
NKX 6.1 reverse	CCGCCAAGTATTTTGTTTGT
NGN3 forward	GCTCATCGCTCTCTATTCTTTTGC
NGN3 reverse	GGTTGAGGCGTCATCCTTTCT
GAPDH forward	GTGGACCTGACCTGCCGTCT
GAPDH reverse	GGAGGAGTGGGTGTCGCTGT
